# Recent Advances in Pharmacotherapy Development for Abdominal Aortic Aneurysm

**DOI:** 10.1155/2012/648167

**Published:** 2012-08-21

**Authors:** Koichi Yoshimura, Hiroki Aoki

**Affiliations:** ^1^Department of Surgery and Clinical Science, Yamaguchi University Graduate School of Medicine, 1-1-1 Minami Kogushi, Ube, Yamaguchi 755-8505, Japan; ^2^Graduate School of Health and Welfare, Yamaguchi Prefectural University, Yamaguchi 753-8502, Japan; ^3^Cardiovascular Research Institute, Kurume University, Kurume, Fukuoka 830-0011, Japan

## Abstract

Abdominal aortic aneurysm (AAA) is a common disease causing segmental expansion and rupture of the aorta with a high mortality rate. The lack of nonsurgical treatment represents a large and unmet need in terms of pharmacotherapy. Advances in AAA research revealed that activation of inflammatory signaling pathways through proinflammatory mediators shifts the balance of extracellular matrix (ECM) metabolism toward tissue degradation. This idea is supported by experimental evidence in animal models that pharmacologic intervention at each pathological step can prevent AAA development. Previously, we identified c-Jun N-terminal kinase (JNK), a pro-inflammatory signaling molecule, as a therapeutic target for AAA. Abnormal activation of JNK in AAA tissue regulates multiple pathological processes in a coordinated manner. Pharmacologic inhibition of JNK tips the ECM balance back towards repair rather than degradation. Interventions targeting signaling molecules such as JNK in order to manipulate multiple pathological processes may be an ideal therapeutic strategy for AAA. Furthermore, the development of biomarkers as well as appropriate drug delivery systems is essential to produce clinically practical pharmacotherapy for AAA.

## 1. Introduction

Abdominal aortic aneurysm (AAA) is a common and fatal disease that is among the top 15 causes of death in elderly men. The incidence of AAA has greatly increased over recent decades, although a recent report suggests that the incidence of AAA may now be declining [1, 2]. Because most AAA patients have no symptoms until the catastrophic event of aneurysmal rupture, the main purpose of treatment is to prevent this rupture, thereby improving prognosis. Patients with large aneurysms are at high risk for rupture and, therefore, are treated by open or endovascular repair. When these surgical treatments are not applicable, an AAA inevitably progresses by gradually increasing its diameter and, concomitantly, the risk of rupture. In addition, close observation is recommended for patients with small aneurysms because of the lack of effective non-surgical treatment options [3]. Therefore, medical treatment, especially pharmacotherapy, for AAA has long been desired.

## 2. Pathological Features of Human AAA

**Figure 1:**
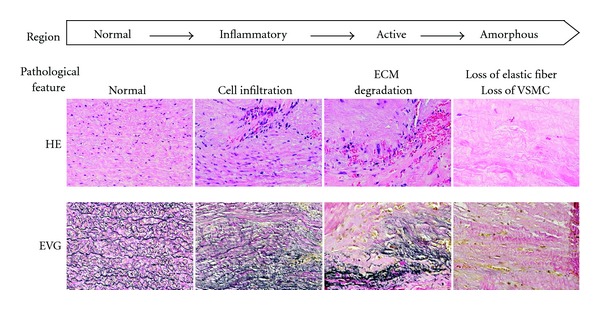
Heterogeneity of the histopathology of human abdominal aortic aneurysm (AAA). Regional heterogeneity within three distinct regions—inflammatory, active, and amorphous—is demonstrated. The order of these distinct regions may correspond to AAA progression from early to advanced phases. ECM: extracellular matrix; VSMC: vascular smooth muscle cell; HE: hematoxylin and eosin; EVG: elastica Van Gieson.

It is generally accepted that an AAA is characterized by chronic inflammation and degradation of the extracellular matrix (ECM) by proteolytic enzymes, such as matrix metalloproteinases (MMPs), leading to segmental dilatation of the aortic wall and eventual rupture with a high mortality rate [4–7]. Importantly, these pathological changes are not distributed homogeneously throughout the aneurysmal wall. We, as well as Curci et al., have pointed out that the histopathology of human AAA exhibits three distinct regions: inflammatory, active, and amorphous [7–10]. These distinct regions are characterized by the cellular components and ECM architecture. The wall of a normal-sized aorta without aneurysmal change shows some intimal thickening and a media composed of well-preserved elastic lamellae with orderly layers of vascular smooth muscle cells (VSMCs), but no inflammatory cell infiltration. The inflammatory region of the vascular wall of an AAA is characterized by a large number of inflammatory cells frequently localized on the adventitial side of the media. The inflammatory infiltrates, including T and B cells, macrophages, mast cells, and neutrophils, secrete proinflammatory mediators and accelerate chronic inflammation. However, elastic lamellae and VSMCs are still preserved in this region. As elastin-degrading enzymes, such as MMP-9, increase and the number of VSMCs, which produce elastin fibers, decreases, there is increased destruction of elastic lamellae. Thus, the area where elastin degradation is actively ongoing is defined as the active region. Finally, most of the maximally dilated area in the walls of a large AAA is characterized by amorphous tissue with abundant fibrocollagenous ECM. The absence of elastic lamellae and VSMCs is particularly striking (Figure 1). Therefore, each distinct region of an AAA wall includes distinct cells and a distinct extracellular environment. An increasing number of studies continue to be performed in basic and translational AAA research, but only a limited number of studies have focused on the regional heterogeneity of AAA. A strategy for developing clinically effective pharmacotherapies based on a better understanding of such heterogeneous molecular processes in human AAA is needed.

## 3. Therapeutic Targets for Treatment of AAA

### 3.1. Overview

**Figure 2:**
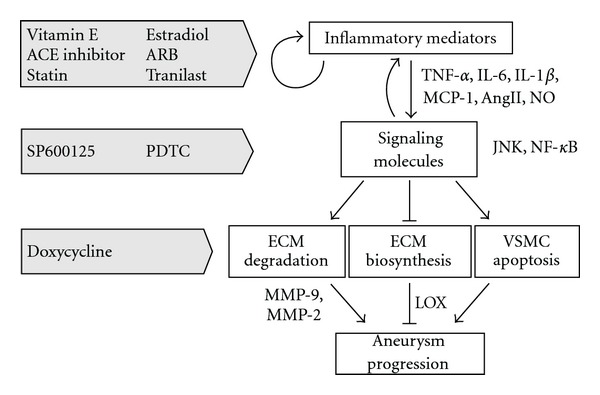
Pathogenesis of abdominal aortic aneurysm (AAA) with potential therapeutic drugs. Intracellular signaling molecules are activated by several types of proinflammatory mediators, while activated signaling pathways also enhance inflammatory mediators. Moreover, the activated signaling molecules shift the balance of extracellular matrix metabolism toward degradation, leading to AAA progression. Pharmacologic agents targeting each aspect of the pathological processes are demonstrated. ACE: angiotensin converting enzyme; ARB: angiotensin II receptor blocker; PDTC: pyrrolidine dithiocarbamate; TNF: tumor necrosis factor; IL: interleukin; MCP: monocyte chemoattractant protein; Ang: angiotensin; NO: nitric oxide; JNK: c-jun N-terminal kinase; NF: nuclear factor; MMP: matrix metalloproteinase; LOX: lysyl oxidase; ECM: extracellular matrix; VSMC: vascular smooth muscle cell.

Activation of proinflammatory signaling pathways through proinflammatory mediators shifts the balance of ECM metabolism towards tissue degradation. Various inflammatory mediators, such as tumor necrosis factor-*α* (TNF-*α*) and monocyte chemoattractant protein-1 (MCP-1), are involved in the pathogenesis of AAA by causing and maintaining the inflammatory response including inflammatory cell infiltration. Intracellular signaling molecules, such as c-Jun N-terminal kinase (JNK) and nuclear factor-*κ*B (NF-*κ*B), are activated by most proinflammatory mediators, while activated signaling pathways enhance expression of proinflammatory mediators, thus propagating a vicious cycle of chronic inflammation. Proinflammatory signaling pathways, in turn, activate ECM degradation enzymes, such as MMP-9 and MMP-2, and simultaneously reduce the expression of ECM synthetic enzymes, such as lysyl oxidase (LOX), thereby causing an overall loss of elastic fibers and AAA progression. This view is supported by accumulating evidence that the development of AAA in animal models can be suppressed by pharmacologic intervention at each step in its molecular pathogenesis. Many reported drugs are effective in suppressing proinflammatory mediators, modulating the intracellular signaling pathways, and inhibiting ECM degradation (Figure 2). Here, we summarize recent advances in pharmacotherapeutic strategies for AAA, which have been examined primarily in animal models (Table 1).

### 3.2. Proinflammatory Mediators

#### 3.2.1. *α*-Tocopherol

Human AAA is associated with a local increase in the production of reactive oxygen species, which may act as proinflammatory mediators. *α*-Tocopherol (vitamin E), a lipid-soluble antioxidant, was found to significantly attenuate the formation of AAA in two animal models [11, 13]. In a study using the angiotensin II- (Ang II-) induced AAA model, treatment with vitamin E decreased the 8-isoprostane content (a marker of oxidative stress) and reduced macrophage infiltration [11]. These findings indicate that oxidative stress may play a significant role in these AAA models. However, a large randomized study reported that long-term supplementation with vitamin E did not reduce hospital admissions for elective AAA repair or reduce the rate of AAA rupture [12].

#### 3.2.2. 17*β*-Estradiol

 Females are much less susceptible to AAA than males [9]. This gender difference is lost after menopause, suggesting that reproductive hormones, including estrogens, may play protective roles against the development of AAA. Ailawadi et al. reported that rats treated with 17*β*-estradiol exhibited less macrophage infiltrate and lower MMP-9 levels and developed smaller aneurysms after elastase infusion compared to controls [14]. Treatment with 17*β*-estradiol also reduced expression of MCP-1, activity of NF-*κ*B, and aneurysm size in an Ang II-induced AAA model [15]. These data provide evidence of gender-related differences in AAA development, which may reflect an estrogen-mediated reduction in proinflammatory mediators and MMP-9.

#### 3.2.3. Inhibitors of Renin-Angiotensin System (RAS)

 Angiotensin-converting enzyme (ACE) inhibitors are widely used in the treatment of hypertension, congestive heart failure, and other cardiovascular disorders. Liao et al. reported the effects of ACE inhibitors on the development of AAA created by elastase infusion in rats. They examined three drugs—captopril, lisinopril, and enalapril. All three prevented the development of AAA and attenuated the degradation of medial elastin without diminishing the inflammatory response [16]. Interestingly, the aneurysm-suppressing effects of ACE inhibitors were dissociated from their effects on systemic hemodynamics. Fujiwara et al. reported the effects of an angiotensin II type 1 (AT1) receptor antagonist, valsartan, on development of AAA created by elastase infusion in rats. Treatment with valsartan prevented AAA development and infiltration of macrophages, while suppressing NF-*κ*B activation and MMP-9 expression [19]. Moreover, a case-control study by Hackam et al. demonstrated that administration of ACE inhibitors was associated with a reduced risk of AAA rupture, though AT1 receptor blockers did not exhibit a significant benefit in terms of preventing AAA rupture [17]. In contrast, Sweeting et al. reported an association of ACE inhibitors with increased AAA expansion [18]. Thus, evidence is inconsistent for the RAS as a therapeutic target in human AAA.

#### 3.2.4. Statins

 A class of cholesterol-lowering drugs, 3-hydroxy-3-methylglutaryl coenzyme A (HMG-CoA) reductase inhibitors, also known as statins, have gained a great deal of attention because of their pleiotropic effects, which may be beneficial in various vascular diseases [50–52]. Steinmetz et al. reported that simvastatin suppressed aneurysm formation in the elastase infusion model in both C57BL/6 wildtype and hypercholesterolemic *ApoE* ^−/−^ mice [28]. Importantly, treatment with simvastatin had no effect on serum cholesterol levels in either normal or hypercholesterolemic mice, suggesting that the benefit of simvastatin to aneurysm development is independent of its cholesterol-lowering effect. Kalyanasundaram et al. also demonstrated that simvastatin suppressed aneurysm formation in an elastase-induced AAA model in rats and reduced protein levels of MMP-9 and NF-*κ*B [29]. Transcriptome analysis provided evidence that simvastatin treatment downregulated proinflammatory mediators in the aortic wall, including IL-1, TNF-*α*, inducible nitric oxide synthase (iNOS), and several chemokines. Shiraya et al. reported the effects of atorvastatin on development of AAA created by elastase infusion in rats. Treatment with atorvastatin prevented AAA development and macrophage infiltration into the aortic wall, while suppressing MMP-9 and MCP-1 secretion [30]. In addition, we and others reported that statins inhibit the secretion of MMP-9 and MCP-1 in the walls of human AAA in vitro and in vivo [20–23]. Several clinical studies demonstrated an association between statin administration and decreased AAA growth [24–27]. However, the beneficial effect of statins has not been confirmed in large clinical trials [18, 31, 32]. On the other hand, there seems to be consistent evidence that statin therapy improves perioperative and postoperative outcome after AAA repair [53, 54].

#### 3.2.5. Mast Cell Stabilizer

Mast cells are one of the inflammatory cell types found in the walls of human AAA and are likely to play a role in its pathogenesis because activation of mast cells leads to the release of proinflammatory cytokines, such as IL-6 and IFN-*γ*. Sun et al. demonstrated that mast cell activation induced by compound 48/80, a mast cell degranulation agent, increased AAA expansion induced by elastase infusion in mice, whereas disodium cromoglycate (DSCG), a mast cell stabilizer, significantly reduced AAA expansion [33]. In addition, the inhibition of AAA development by DSCG corresponded to preservation of the elastic lamina, decreased infiltration of mast cells and macrophages, and reduced IFN-*γ*, IL-6, and MMP activity. Tsuruda et al. also reported that pharmacological intervention with tranilast, another inhibitor of mast cell degranulation, attenuated AAA development in two rodent models [34]. Although these data raise considerable interest in the use of mast cell stabilizing drugs for the treatment of patients with AAA, no clinical trial results have been reported revealing their efficacy in reducing AAA expansion.

### 3.3. Intracellular Signaling Pathways

Recently, intracellular signaling pathways have attracted attention as therapeutic targets of AAA. We and others demonstrated that the pharmacologic inhibition of certain signaling pathways is effective in treating experimental AAA, including the Rho/Rho-kinase, NF-*κ*B, and JNK pathways [35, 36, 55].

Wang et al. reported that treatment with fasudil, a Rho-kinase inhibitor, resulted in reduction of both the incidence and severity of Ang II-induced AAA [55]. They also demonstrated that fasudil treatment inhibited both VSMC apoptosis and proteolysis by MMP-9 and MMP-2. Parodi et al. studied the effects of NF-*κ*B inhibition by pyrrolidine dithiocarbamate (PDTC) on the development of elastase-induced AAA [35]. Treatment with PDTC suppressed NF-*κ*B activity, expressions of MMP-9 and proinflammatory cytokines, and formation of AAA in mice. These results were further supported by other studies demonstrating the suppression of experimental AAA by NF-*κ*B/Ets decoy oligonucleotide [56, 57].

**Figure 3:**
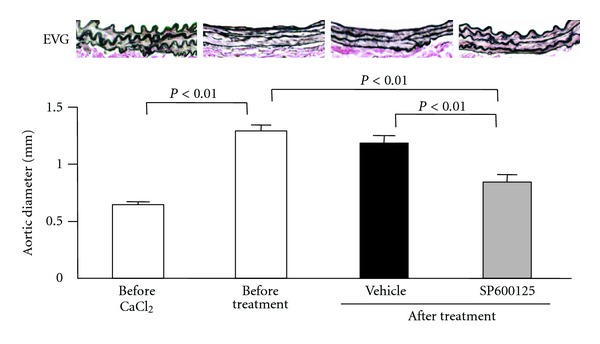
Regression of abdominal aortic aneurysm (AAA) with c-Jun N-terminal kinase (JNK) inhibitor in a mouse model. Six weeks after stimulation of mouse aorta with CaCl_2_, the AAA model was established in association with elastic lamellae disruption and increased aortic diameter. After AAA establishment, pharmacologic treatment with SP600125, a JNK inhibitor, was initiated. After six weeks of SP600125 treatment, there was a significant reduction in aneurysmal size compared with vehicle treatment as well as before treatment. The regression of AAA was accompanied by a repair of tissue architecture. EVG: elastica Van Gieson (modified from Yoshimura et al. [36]).

We identified JNK as a therapeutic target for AAA treatment by screening the activation status of signaling molecules in human AAA tissue [5, 36, 37, 58]. JNK is a key regulator of AP-1, which is a critical transcriptional regulator of MMP-9 and various proinflammatory cytokines. SP600125, a specific JNK inhibitor, completely prevented the development of calcium-induced AAA in mice and significantly reduced MMP-9, macrophage infiltration, and the preservation of elastic lamellae. Moreover, treatment with SP600125 after the establishment of AAA formation resulted in a striking reduction of the aneurysm diameter and restored the once-disrupted elastic lamellae, indicating that JNK inhibition enhances the repair of tissue architecture (Figure 3). In addition, treatment with SP600125 resulted in reduced aneurysm diameters of Ang II-induced AAA in *ApoE* ^−/−^ mice. These data demonstrated for the first time that pharmacological treatment causes regression of an established AAA in animal models [36]. SP600125 also suppressed the secretion of MMP-9 and MMP-2 in the walls of human AAA in ex vivo culture. Thus, pharmacological inhibition of JNK is thought to be a potentially effective therapeutic option for the treatment of AAA [59, 60].

### 3.4. Enzymes for ECM Metabolism

The development, progression, and rupture of AAA are closely associated with connective tissue destruction. In particular, the biophysical properties of the aneurysmal aorta are largely due to a loss of medial and adventitial elastin. It is widely accepted that elastolytic MMPs, particularly MMP-9 and MMP-2, cause degradation of ECM, thereby leading to the development of AAA [5, 9, 44, 61]. Petrinec et al. first reported that treatment with doxycycline, a tetracycline derivative, caused a significant reduction in the incidence of AAA induced by elastase infusion in rats [38]. They also observed that doxycycline reduced MMP-9 production and prevented the destruction of elastic lamellae without decreasing inflammatory cell infiltration. Their results were further supported in various experimental conditions [39–41, 44–49]. Because of the promising results in these animal studies, the effect of doxycycline on human AAA has been investigated. Curci et al. demonstrated that preoperative treatment with doxycycline decreased MMP-9 expression in aneurysm tissue [42]. Mosorin et al. first published a randomized trial of doxycycline in patients with small AAA [62]. They showed that the expansion rate of AAA treated with doxycycline (1.5 mm/year) was lower than those treated with placebo (3.0 mm/year), but the difference did not reach statistical significance. Baxter et al. conducted a clinical trial evaluating the effect of doxycycline in patients with small AAA [43]. They demonstrated that doxycycline was safe and well tolerated and was associated with a gradual reduction of plasma MMP-9 levels. In addition, Lindeman et al. reported that doxycycline reduced inflammation in the walls of human AAA by inhibiting infiltration of neutrophils and cytotoxic T cells [63]. Thus, MMP inhibitors, especially doxycycline, are the most extensively tested pharmacotherapy for AAA treatment in both animal models and clinical trials. However, further clinical studies are still needed to confirm the beneficial effects of MMP inhibitors on the growth of human AAA.

ECM biosynthetic enzymes, such as LOX, are essential for stabilization of collagen and elastin fibers. A critical role of ECM biosynthesis in aortic wall integrity was demonstrated by the observation that disruption of the LOX gene leads to aneurysm formation and aortic rupture [64]. Reduced LOX expression in experimental AAA has also been reported [36, 40]. Indeed, we demonstrated that adenoviral expression of LOX ameliorated experimental AAA progression, indicating that impaired ECM biosynthesis plays a critical role in the development of AAA [36]. More interestingly, we found that enhancement of LOX activity not only stabilized the ECM but also reduced inflammatory responses, including MCP-1 secretion, macrophage infiltration, and JNK activation, thereby preventing AAA progression in mice [65]. Therefore, enhancement of LOX activity may represent a new therapeutic target for the treatment of AAA.

## 4. Future Directions

Since AAA is predominately localized to a limited site on the aorta, local delivery of pharmacologic agents, which would increase therapeutic efficacy and reduce systemic side effects, is a reasonable approach. The efficacy of this therapeutic option was previously reported using doxycycline and rodent models of AAA [41, 45]. The combination of local drug delivery with other interventions, such as endovascular stent grafting, is one possibility. Because ongoing aortic wall degeneration and subsequent failure of aneurysm exclusion are the major concern, after endovascular repair, adjuvant pharmacotherapy would provide an ideal solution for these concerns. Indeed, patent applications claiming a device composed of stent graft and drug delivery system have been filed by us and others [66, 67]. Further progress with drug delivery systems could advance the development of less invasive therapeutic strategies for the treatment of AAA.

Today, several pharmacologic agents targeting various pathological components, including ECM degrading enzymes, proinflammatory mediators, and intracellular signaling pathways, are documented to be effective at least in AAA animal models. Therefore, it seems very possible for us to manipulate some aspects of the biological process, such as the enhancement of proinflammatory mediators, the activation of intracellular signaling pathways, and the shift of ECM metabolism toward degradation. The challenge is to diagnose the predominant aspect in a given patient, or even in a given area of the AAA tissue. Another challenge is monitoring tissue response during pharmacotherapy to optimize the therapeutic regimen to fit individual patients' needs. To this end, it is necessary to identify biomarkers that accurately reflect the biological activity in AAA, as proposed by Golledge and Powell [68]. Several circulating biomarkers for AAA have emerged as candidates [69, 70]. However, they have yet to be tested in a large standardized study to reveal their potential utility in the prediction of AAA progression as well as in the diagnosis of disease activity.

## 5. Conclusions

In past decades, several studies have identified classes of drugs that can ameliorate various pathological activities in AAA. Human AAA will be treatable by pharmacological therapy if we can optimize the therapeutic regimen for the individual patient with the aid of biomarkers. Despite current improvements in therapeutic options, including open surgical and endovascular aneurysm repairs, nonsurgical prevention of AAA growth and rupture has yet to be achieved. Once established, pharmacotherapy could play a crucial role in the management of patients with AAA.

## Figures and Tables

**Table 1:** Pharmacotherapy for abdominal aortic aneurysm in animal models.

Target	Drug	Model	Mechanism of AAA inhibition	Effects in human AAA
Oxidative stress	*α*-Tocopherol (vitamin E)	ATII/*ApoE * ^−/−^ mice [11]	ROS ↓, macrophage infiltration ↓	No effect on growth [12]
		Elastase/rat [13]		

Estrogen receptor	17*β*-estradiol	Elastase/rat [14]	Macrophage infiltration ↓, MCP-1 ↓,	No evidence
		ATII/*ApoE * ^−/−^ mice [15]	NF-*κ*B activity ↓, MMP-9 ↓, preserved elastin	

RAS	ACE inhibitor (captopril, lisinopril, and enalapril)	Elastase/rat [16]	Preserved elastin	Rupture risk ↓ [17] growth ↑ [18]
	ARB (valsartan)	Elastase/rat [19]	Macrophage infiltration ↓, NF-*κ*B activity ↓, MMP-9 ↓	No effect on rupture risk [17]

Mevalonate pathway	Statin (simvastatin and atorvastatin)	Elastase/mice	Macrophage infiltration ↓, IL-1 ↓, MCP-1 ↓, NF-*κ*B activity ↓, MMP-9 ↓,	MCP-1 ↓, MMP-9 ↓ [20–23] growth ↓ [24–27]
		Elastase/*ApoE * ^−/−^ mice [28]		
		Elastase/rat [29, 30]	Preserved elastin	No effect on growth [18, 31, 32]

Mast cell	DSCG [33]	Elastase/mice	Mast cell and macrophage infiltration ↓, IFN-*γ*↓, IL-6 ↓, MMP activity ↓, preserved elastin	No evidence
	Tranilast [34]	CaCl_2_/rat		
		ATII/*ApoE * ^−/−^ mice		

NF-*κ*B	PDTC [35]	Elastase/mice	Cellular infiltration ↓, IL-1*β*↓, IL-6 ↓, NF-*κ*B activity ↓, MMP-9 ↓, preserved elastin	No evidence

JNK	SP600125 [36]	CaCl_2_/ mice	Macrophage infiltration ↓, MMP-9 ↓, preserved elastin, regression of established AAA	MMP-9 ↓, TIMP-3 ↑ [36, 37]
		ATII /*ApoE * ^−/−^ mice		

MMP	Doxycycline	Elastase/rats [38–41]	MMP-9 ↓, preserved elastin	MMP-9 ↓ [42, 43]
		Elastase/mice [44, 45]		
		CaCl_2_/mice [46]		
		ATII/*ApoE * ^−/−^ mice [47, 48]		
		Thioglycolate plus plasmin/rats [49]		

AAA: abdominal aortic aneurysm; ATII: angiotensin II; ROS: reactive oxygen species; MMP: matrix metalloproteinase; NF: nuclear factor; MCP: monocyte chemoattractant protein; RAS: renin-angiotensin system; ACE: angiotensin converting enzyme; ARB: angiotensin receptor blocker; IL: interleukin; DSCG: disodium cromoglycate; IFN: interferon; PDTC: pyrrolidine dithiocarbamate; JNK: c-Jun N-terminal kinase; TIMP: tissue inhibitor of metalloproteinase.
